# Supplementary material for: Examining the Role of Extrachromosomal DNA in 1,216 Lung Cancers
Source: bioRxiv. 2025 Jul 24:2025.06.03.657117. Originally published 2025 Jun 7. Preprint. [Version 2] doi: 10.1101/2025.06.03.657117 (PMC12157361; doi:10.1101/2025.06.03.657117)

Supplementary Fig. 1

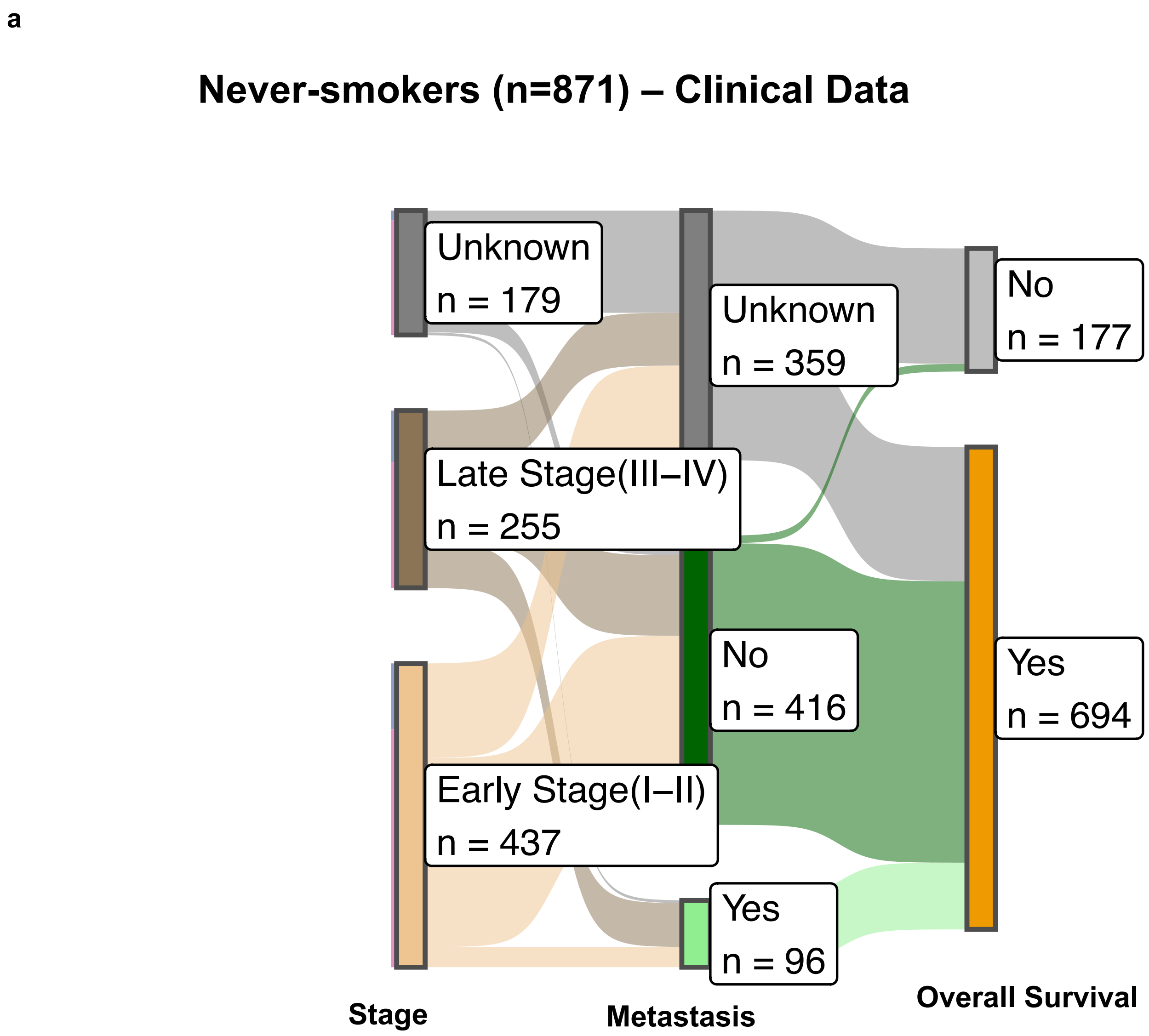

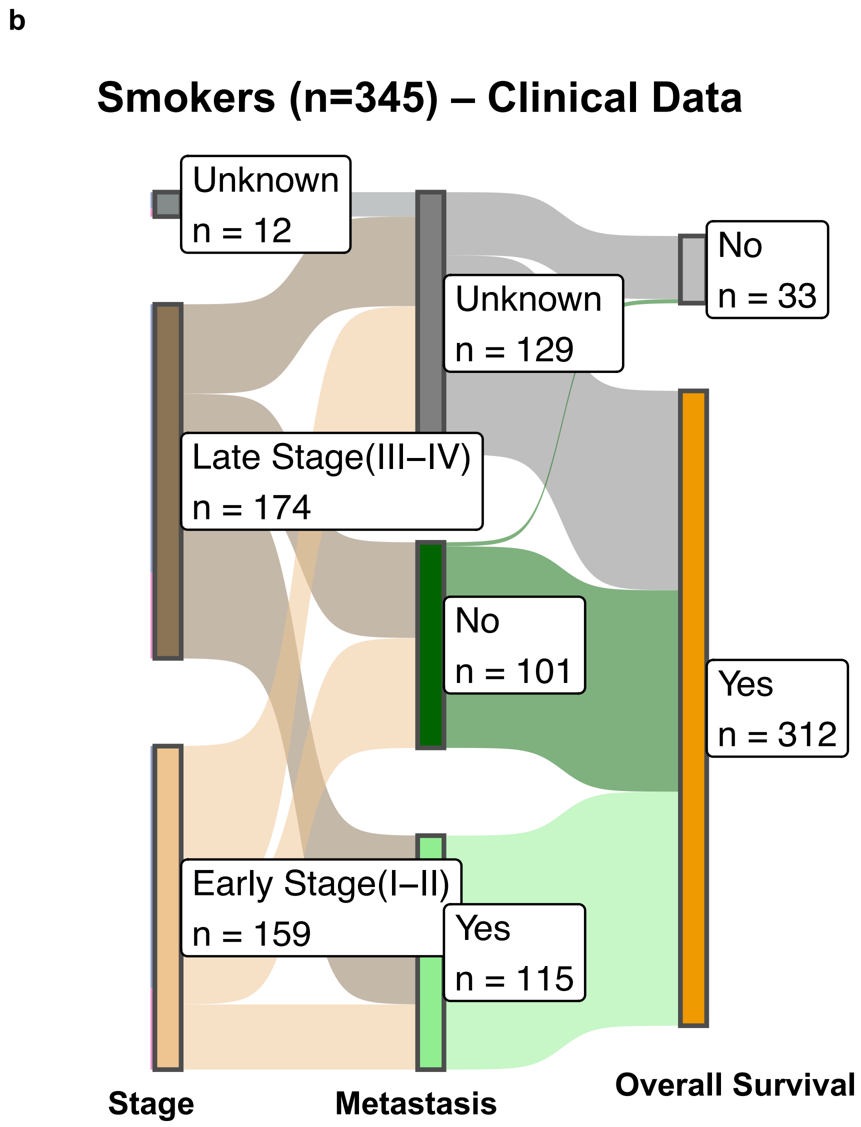

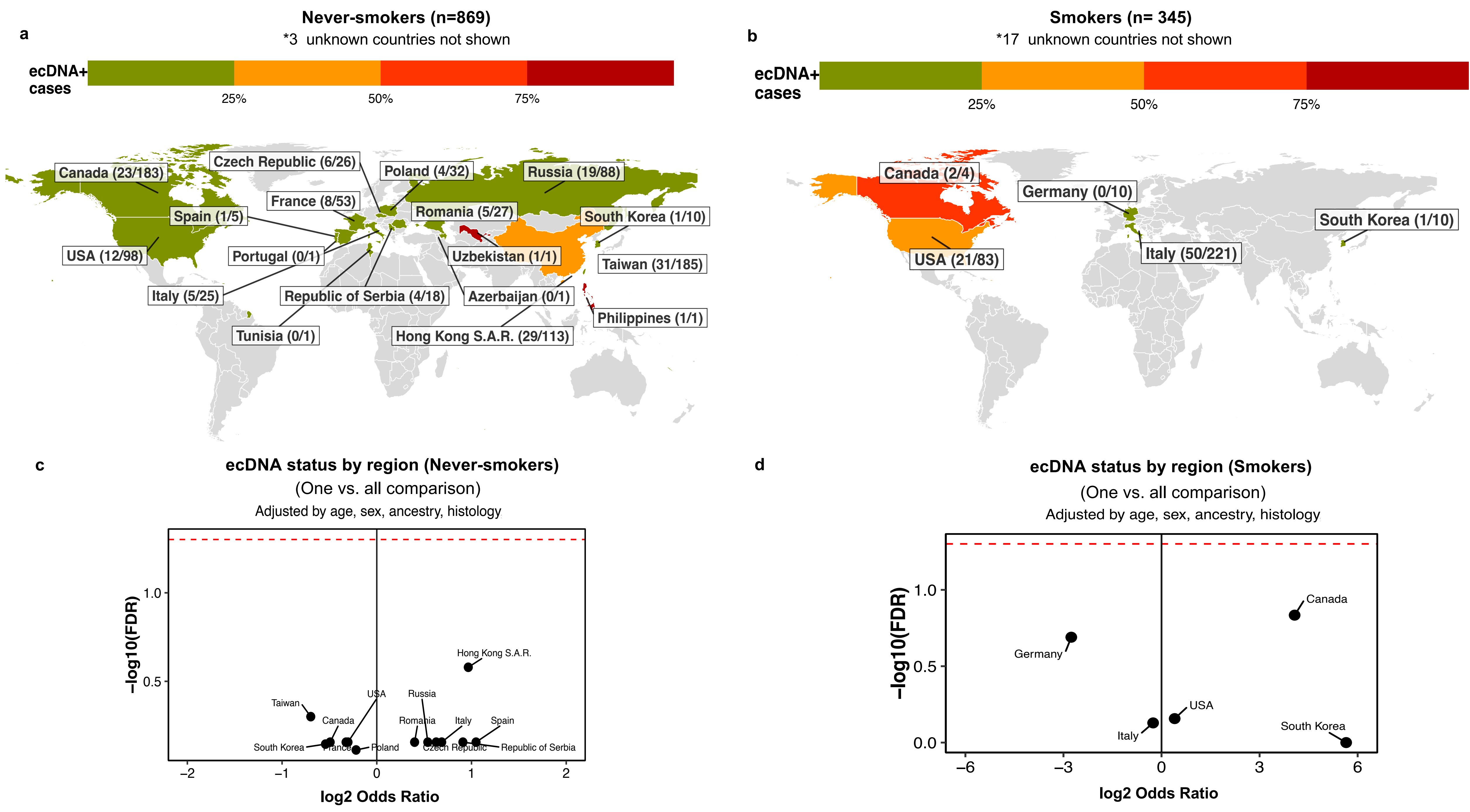
a
Never-smokers (n=869)
*3 unknown countries not shown
ecDNA+ cases
25%
50%
75%
Canada (23/183)
Czech Republic (6/26)
Poland (4/32)
Russia (19/88)
France (8/53)
Spain (1/5)
Romania (5/27)
South Korea (1/10)
USA (12/98)
Portugal (0/1)
Uzbekistan (1/1)
Taiwan (31/185)
Italy (5/25)
Republic of Serbia (4/18)
Azerbaijan (0/1)
Philippines (1/1)
Tunisia (0/1)
Hong Kong S.A.R. (29/113)
b
Smokers (n= 345)
*17 unknown countries not shown
ecDNA+ cases
25%
50%
75%
Canada (2/4)
Germany (0/10)
South Korea (1/10)
Italy (50/221)
USA (21/83)
c
ecDNA status by region (Never-smokers)
(One vs. all comparison)
Adjusted by age, sex, ancestry, histology
−log10(FDR)
log2 Odds Ratio
Hong Kong S.A.R.
Taiwan
USA
Russia
Canada
Romania
Italy
Spain
South Korea
France
Poland
Czech Republic
Republic of Serbia
d
ecDNA status by region (Smokers)
(One vs. all comparison)
Adjusted by age, sex, ancestry, histology
−log10(FDR)
log2 Odds Ratio
Canada
Germany
USA
Italy
South Korea

Supplementary Fig. 3

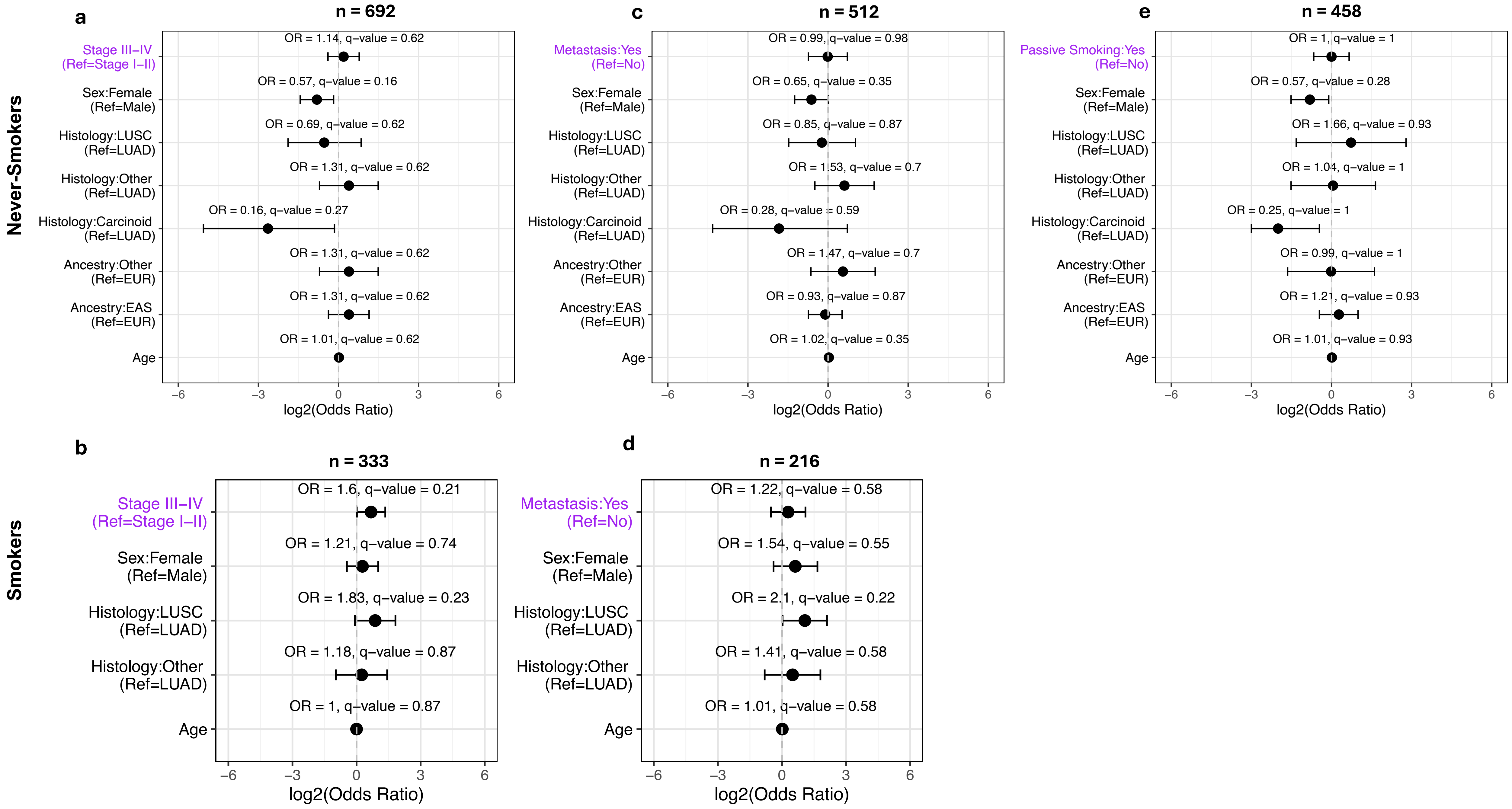

Supplementary Fig. 4

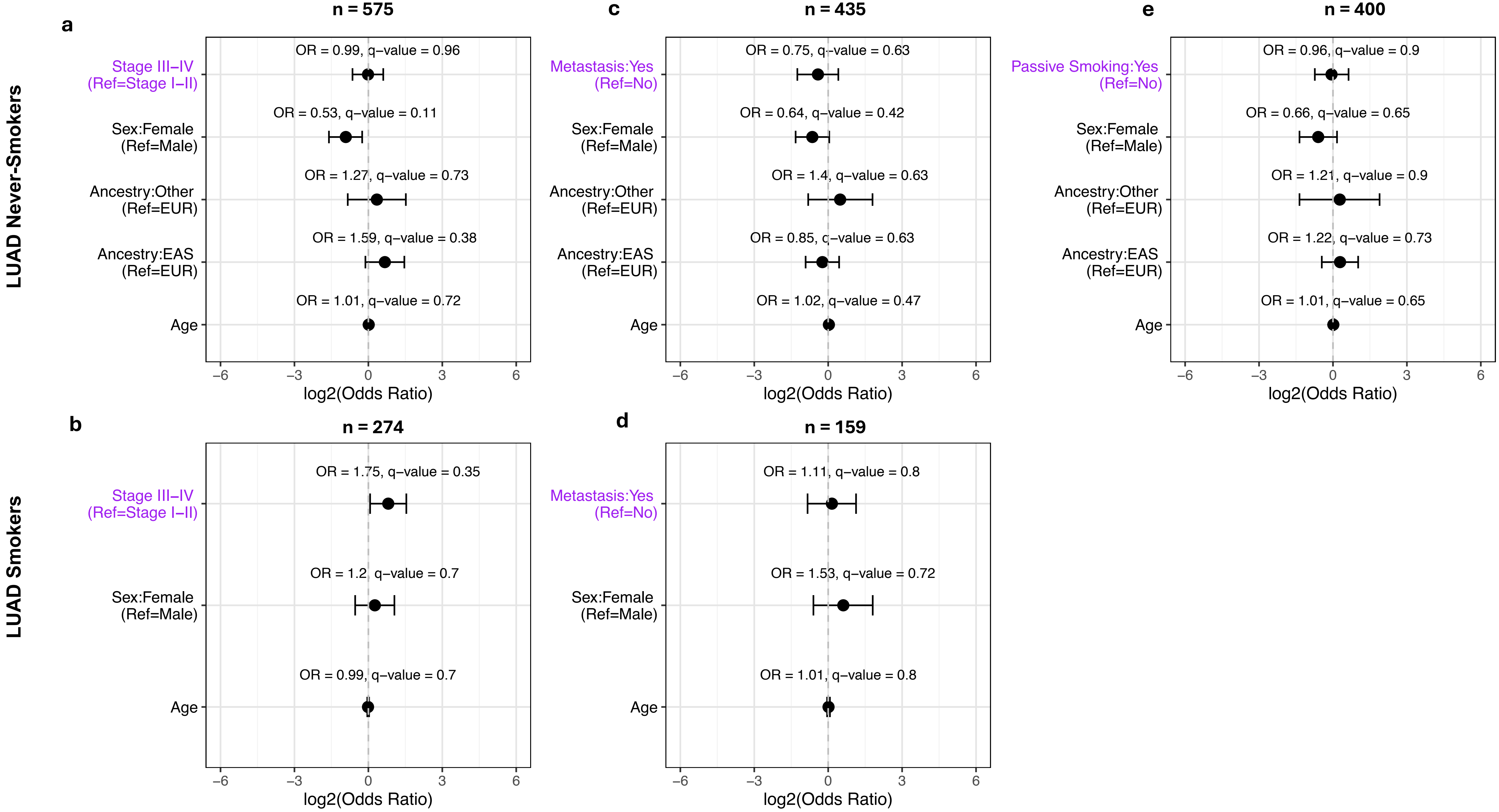

Supplementary Fig. 5

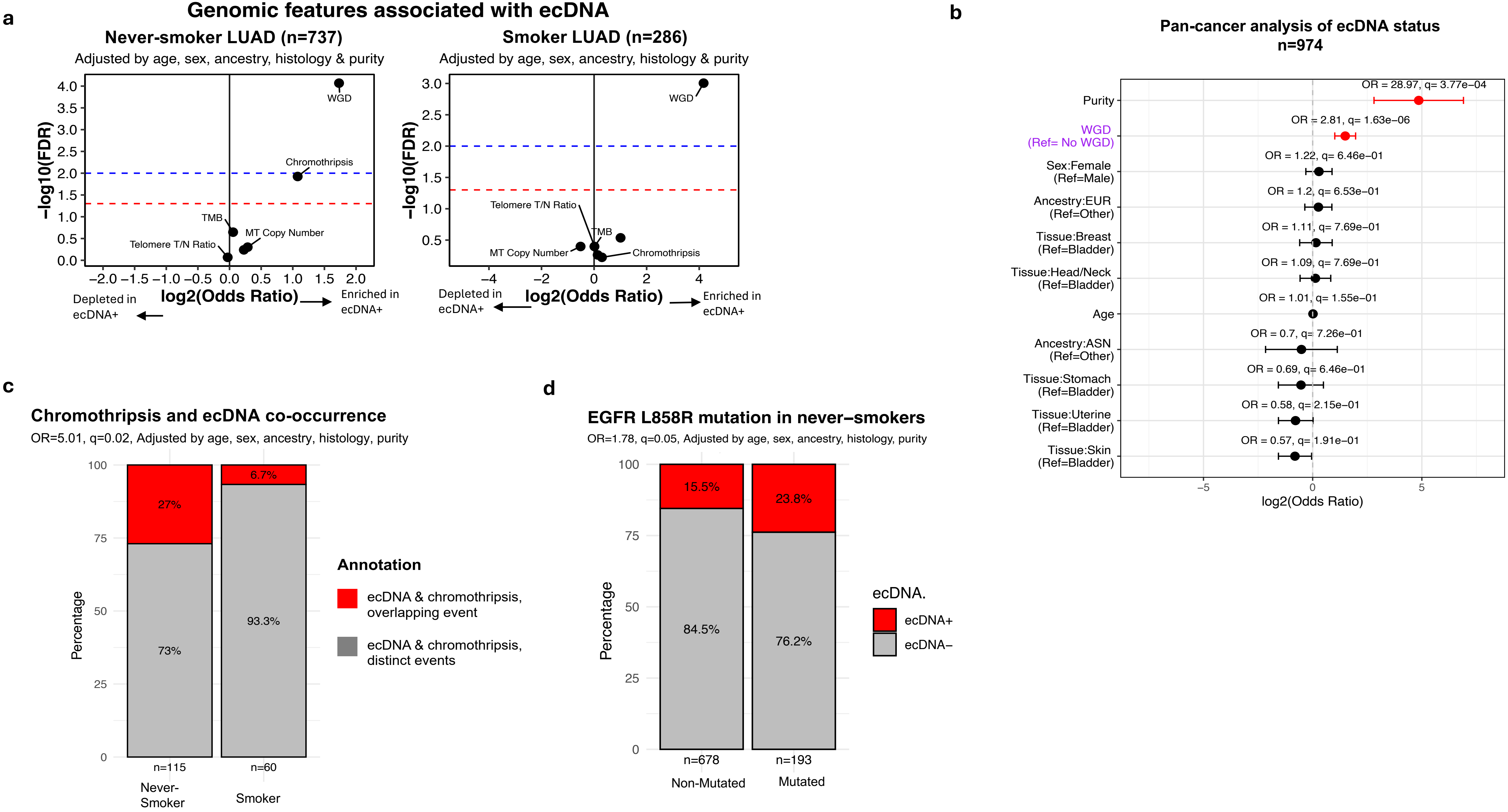

Supplementary Fig. 6

Mutational signatures associated with ecDNA

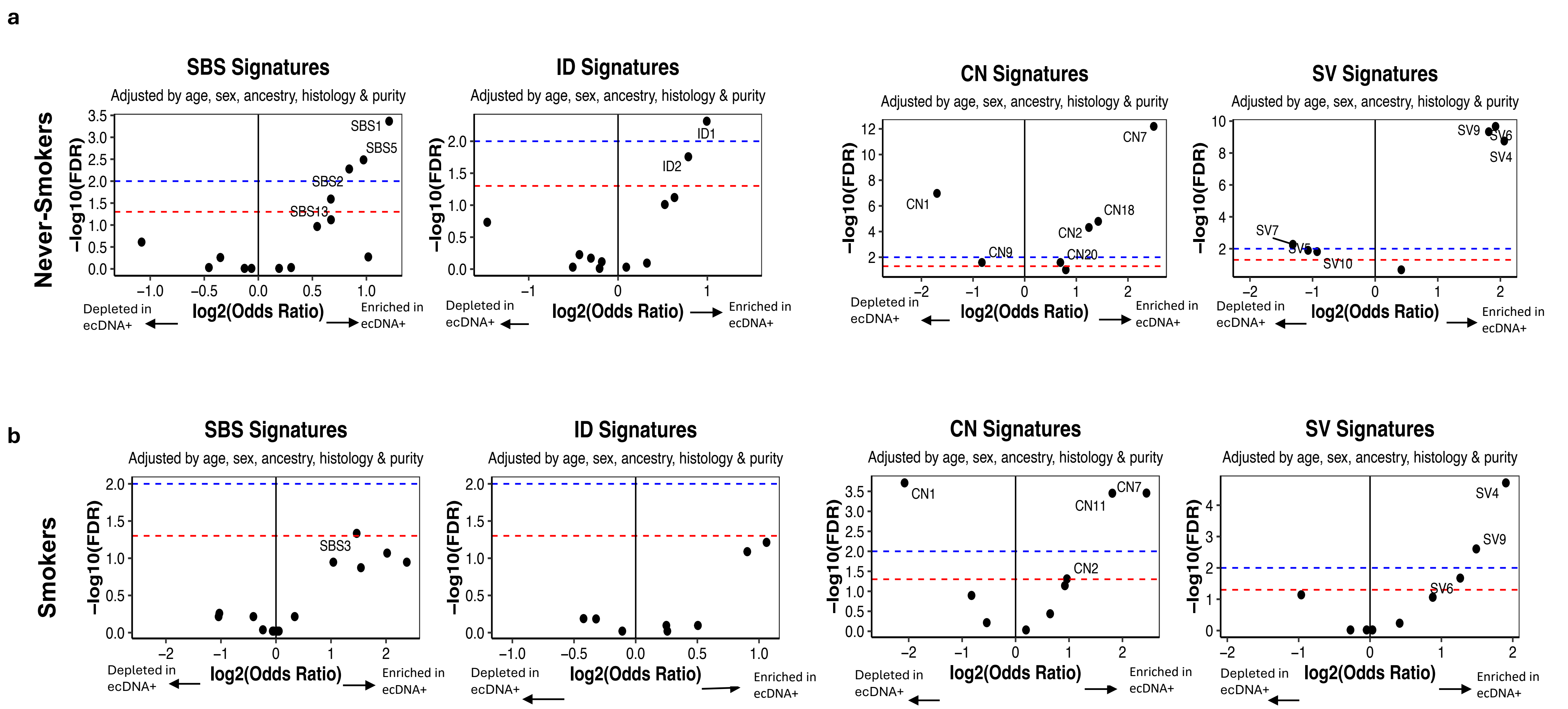

Supplementary Fig. 7

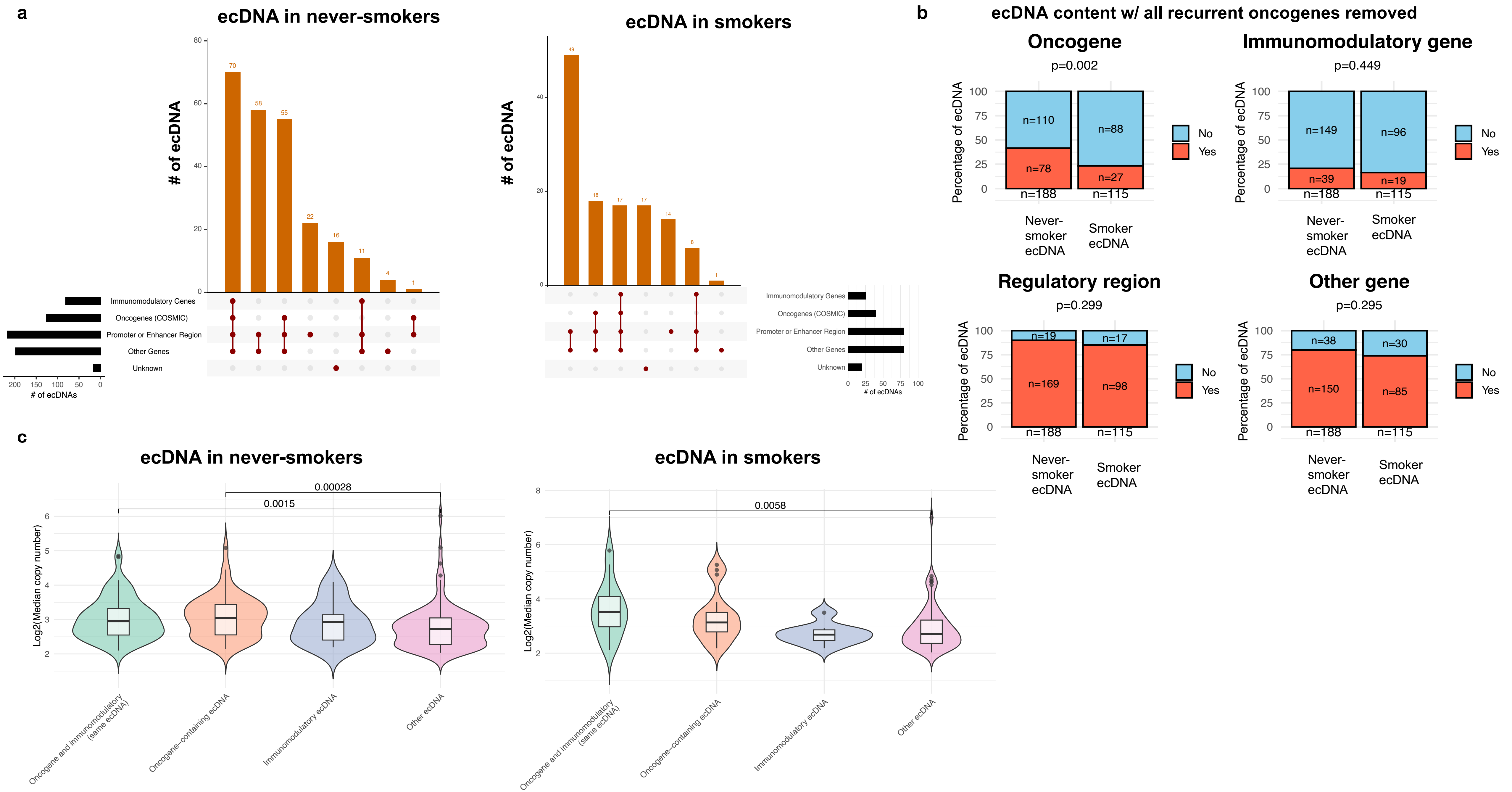

Supplementary Fig. 8

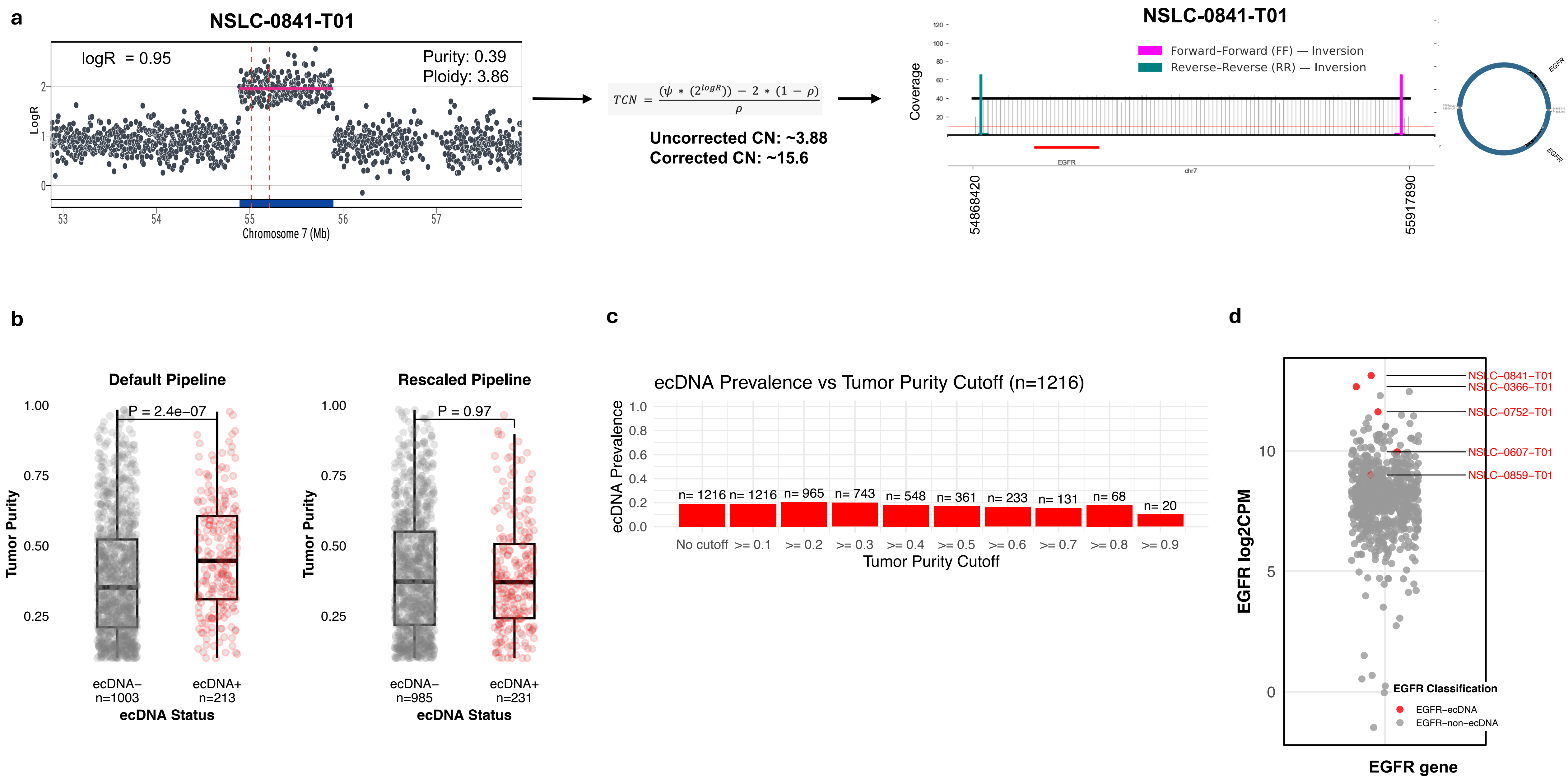

Supplement: Supplement 2 — Supplementary Fig 1. Additional cohort information. Sankey diagrams showing the numbers of samples with clinical variables (stage, metastasis) as well as survival data available for a) LCINS and b) LCSS Supplementary Fig 2. Distribution of ecDNA harboring lung cancers in LCINS and LCSS by country. Maps showing the worldwide prevalence of lung cancers harboring ecDNA (ecDNA+) by country for the a) LCINS cohort and b) LCSS cohort. Volcano plot of a multivariate one-vs-all logistic regression model for each country, with ecDNA status as the outcome, for c) LCINS and d) LCSS. Supplementary Fig 3. Analysis of the association between stage, metastasis, and passive smoking with ecDNA status in all LCINS and LCSS. Forest plots of logistic regression model with ecDNA status as the outcome and the variables for stage in a) LCINS and b) LCSS; metastasis in c) LCINS and d) LCSS; and passive smoking in e) LCINS. The total number of samples with information for all variables is indicated above each plot. Supplementary Fig 4. Analysis of the association between stage, metastasis, and passive smoking with ecDNA status in lung adenocarcinomas. Forest plots of logistic regression model with ecDNA status as the outcome and the variables for stage in a) LCINS and b) LCSS; metastasis in c) LCINS and d) LCSS; and passive smoking in e) LCINS. The total number of samples with information for all variables is indicated above each plot. Supplementary Fig 5. Genomic features associated with the presence of ecDNA. a) Volcano plot of logistic regression models of genomic features in association with ecDNA status for lung adenocarcinoma in LCINS (left) and lung adenocarcinoma in LCSS (right). In all volcano plots, the x-axes reflect the log2 odds ratio, and the y-axes correspond to the log10 FDR q-value. An FDR q-value threshold of 0.05 is indicated with the dashed red line, an FDR q-value threshold of 0.01 is indicated with the dashed blue line. b) Forest plot of multivariate logistic regressio [file media-2.pdf]
