# Supplementary material for: Examining the Role of Extrachromosomal DNA in 1,216 Lung Cancers
Source: bioRxiv. 2025 Jun 7:2025.06.03.657117. Preprint. [Version 1] doi: 10.1101/2025.06.03.657117 (PMC12157361; doi:10.1101/2025.06.03.657117)

bioRxiv preprint doi: https://doi.org/10.1101/2025.06.03.657117; this version posted June 7, 2025. The copyright holder for this preprint (which was not certified by peer review) is the author/funder. This article is a US Government work. It is not subject to copyright under 17 USC 105 and is also made available for use under a CC0 license.

SUPPLEMENTARY FIGURE LEGENDS

Supplementary Fig 1. Additional cohort information. Sankey diagrams showing the numbers of samples with clinical variables (stage, metastasis) as well as survival data available for ***a)*** LCINS and ***b)*** LCSS

Supplementary Fig 2. Distribution of ecDNA harboring lung cancers in LCINS and LCSS by country. Maps showing the worldwide prevalence of lung cancers harboring ecDNA ($ecDNA^+$) by country for the ***a)*** LCINS cohort and ***b)*** LCSS cohort. Volcano plot of a multivariate one-vs-all logistic regression model for each country, with ecDNA status as the outcome, for ***c)*** LCINS and ***d)*** LCSS. ***e)*** Bar plot depicting the prevalence of ecDNA in LCINS *versus* LCSS from the United States. Statistical results from a logistic regression model are shown above.

Supplementary Fig 3. Analysis of the association between stage, metastasis, and passive smoking with ecDNA status in all LCINS and LCSS. Forest plots of logistic regression model

bioRxiv preprint doi: https://doi.org/10.1101/2025.06.03.657117; this version posted June 7, 2025. The copyright holder for this preprint (which was not certified by peer review) is the author/funder. This article is a US Government work. It is not subject to copyright under 17 USC 105 and is also made available for use under a CC0 license.

with ecDNA status as the outcome and the variables for stage in **a)** LCINS and **b)** LCSS; metastasis in **c)** LCINS and **d)** LCSS; and passive smoking in **e)** LCINS. The total number of samples with information for all variables is indicated above each plot.

Supplementary Fig 4. Analysis of the association between stage, metastasis, and passive smoking with ecDNA status in lung adenocarcinomas. Forest plots of logistic regression model with ecDNA status as the outcome and the variables for stage in **a)** LCINS and **b)** LCSS; metastasis in **c)** LCINS and **d)** LCSS; and passive smoking in **e)** LCINS. The total number of samples with information for all variables is indicated above each plot.

Supplementary Fig 5. Genomic features associated with the presence of ecDNA. ***a)*** Volcano plot of logistic regression models of genomic features in association with ecDNA status for lung adenocarcinoma in LCINS (left) and lung adenocarcinoma in LCSS (right). In all volcano plots, the x-axes reflect the $\log_2$ odds ratio, and the y-axes correspond to the $\log_{10}$ FDR q-value. An FDR q-value threshold of 0.05 is indicated with the dashed red line, an FDR q-value threshold of 0.01 is indicated with the dashed blue line. ***b)*** Forest plot of multivariate logistic regression modeling the presence or absence of ecDNA in relation to whole-genome doubling in a pan-cancer dataset comprising four different tumor types from the PCAWG project, adjusted for the covariates of age, purity, sex, and tissue type. All significant associations are indicated in red. ***c)*** Bar plot indicating the proportion of samples that contained overlapping chromothripsis and ecDNA regions (red) and the proportion of all samples that did not contain any overlap (gray) for all samples that had both an ecDNA and chromothripsis event detected. ***d)*** Bar plot showing the proportion of samples with an *EGFR* L858R mutation, stratified by ecDNA status.

bioRxiv preprint doi: https://doi.org/10.1101/2025.06.03.657117; this version posted June 7, 2025. The copyright holder for this preprint (which was not certified by peer review) is the author/funder. This article is a US Government work. It is not subject to copyright under 17 USC 105 and is also made available for use under a CC0 license.

Supplementary Fig 6. Association of mutational signatures with ecDNA status. Volcano plots of logistic regression models of the activities of Single base substitution (SBS), Indel (ID), copy number (CN) and structural variant (SV) signatures in association with ecDNA in lung cancers of ***a)*** LCINS and ***b)*** LCSS. The logistic regression models were adjusted for age, sex, ancestry, histology, and purity. In all volcano plots, the x-axes reflect the $\log_2$ odds ratio, and the y-axes correspond to the $\log_{10}$ FDR q-value. An FDR q-value threshold of 0.05 is indicated with the dashed red line, an FDR q-value threshold of 0.01 is indicated with the dashed blue line.

Supplementary Fig 7. Genomic content of ecDNA

a) Upset plot indicating the number of ecDNA with oncogenes, immunomodulatory genes, promoter or enhancer regions, other genes that are not oncogenes, or unidentified genomic elements (Unknown) or any combination thereof for LCINS and LCSS. **b)** Bar plot indicating the proportion of ecDNA containing a particular genomic element in LCINS vs LCSS. **c)** $\log_2$ median copy number (y-axis) for each class of ecDNA (x-axis) in LCINS and LCSS.

Supplementary Fig 8. Highly sensitive pipeline for ecDNA detection that accounts for tumor purity and ploidy.

a) Schematic depicting the methodology for the rescaled pipeline that considers tumor purity and ploidy. The logR track for the region containing the *EGFR* gene on chr7 and the purity and ploidy computed from Battenberg is indicated in one tumor as example (left). The exact location of the *EGFR* gene is shown by the dashed red lines. The equation that simultaneously accounts

bioRxiv preprint doi: https://doi.org/10.1101/2025.06.03.657117; this version posted June 7, 2025. The copyright holder for this preprint (which was not certified by peer review) is the author/funder. This article is a US Government work. It is not subject to copyright under 17 USC 105 and is also made available for use under a CC0 license.

for sample purity and ploidy when computing the total copy number is shown, as well as the total copy number estimate when assuming a sample purity of 1.0 and ploidy of 2.0 (Uncorrected CN) *vs*. considering the purity and ploidy (Corrected CN). The Sashimi plot indicates the structural variants (colored arcs) and smoothed coverage around the *EGFR* region, which was annotated as ecDNA by AmpliconClassifier. **b)** Distribution of tumor purities for samples with an ecDNA annotation for both the default (left) and rescaled (right) pipelines. P-value for a Mann-Whitney test between $ecDNA^+$ and $ecDNA^-$ samples is indicated. The total number of samples in each group is also indicated. **c)** The ecDNA prevalence (y-axis), which is the proportion of $ecDNA^+$ samples over the total number of samples, for all possible tumor purity cutoffs applied to samples (x-axis). The total number of samples available for analysis after each tumor purity cutoff is applied is also indicated. **d)** Gene expression (log_2 CPM) of the EGFR gene for all samples with RNA-seq data available. LCINS samples with EGFR-ecDNA are indicated in red.

Supplementary Data Fig. 1

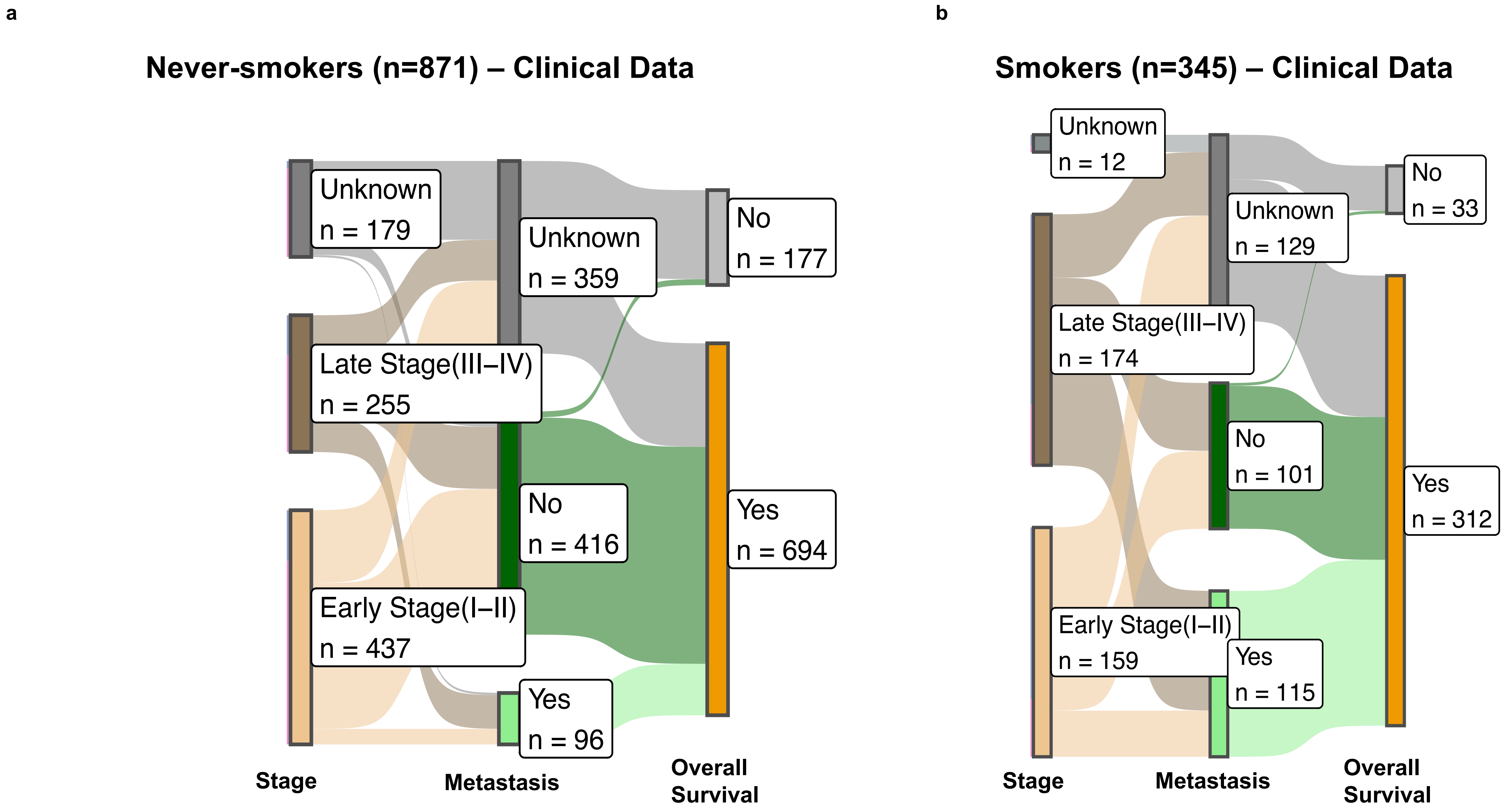

Supplementary Data Fig. 2

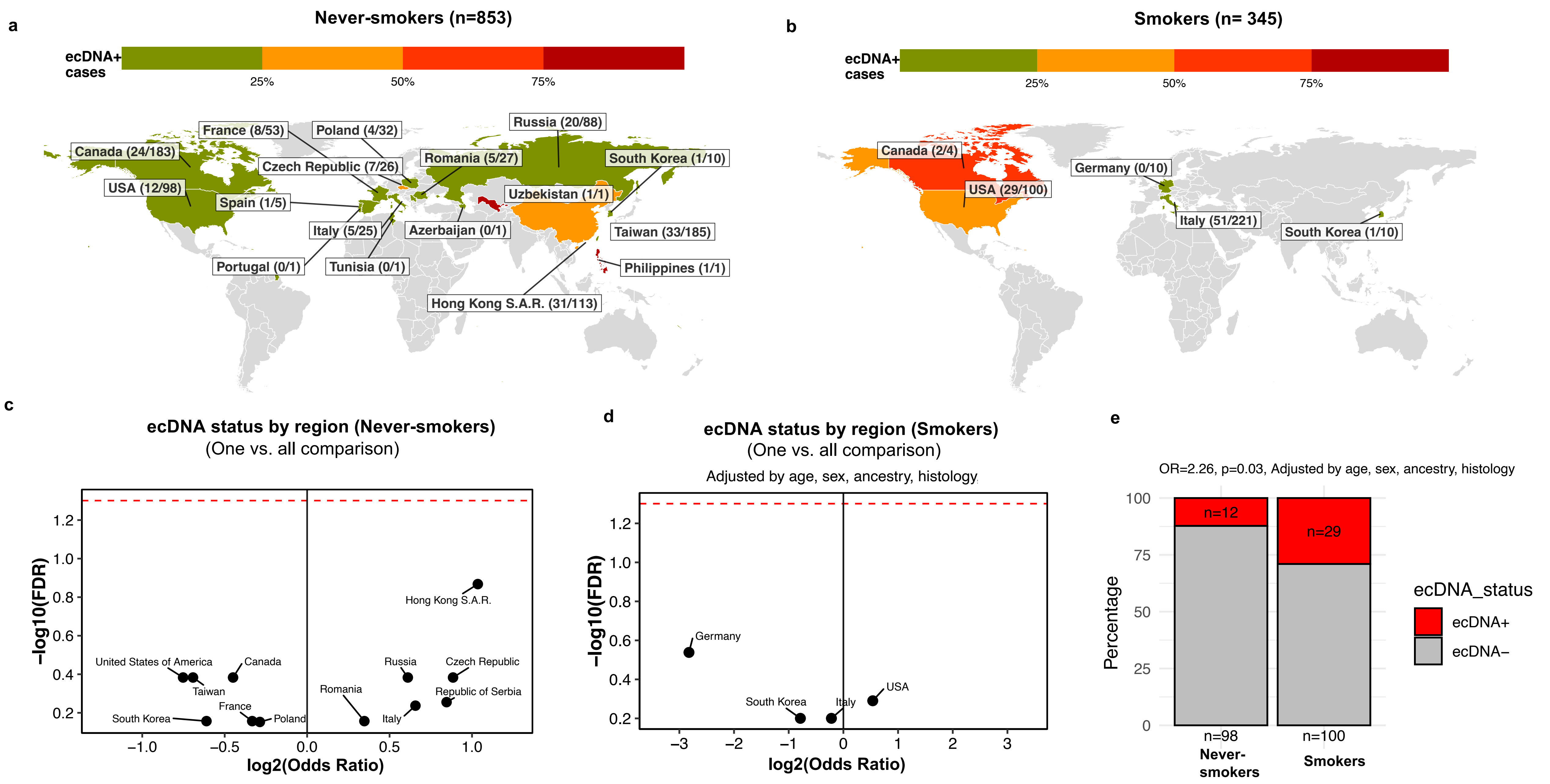

Supplementary Data Fig. 3

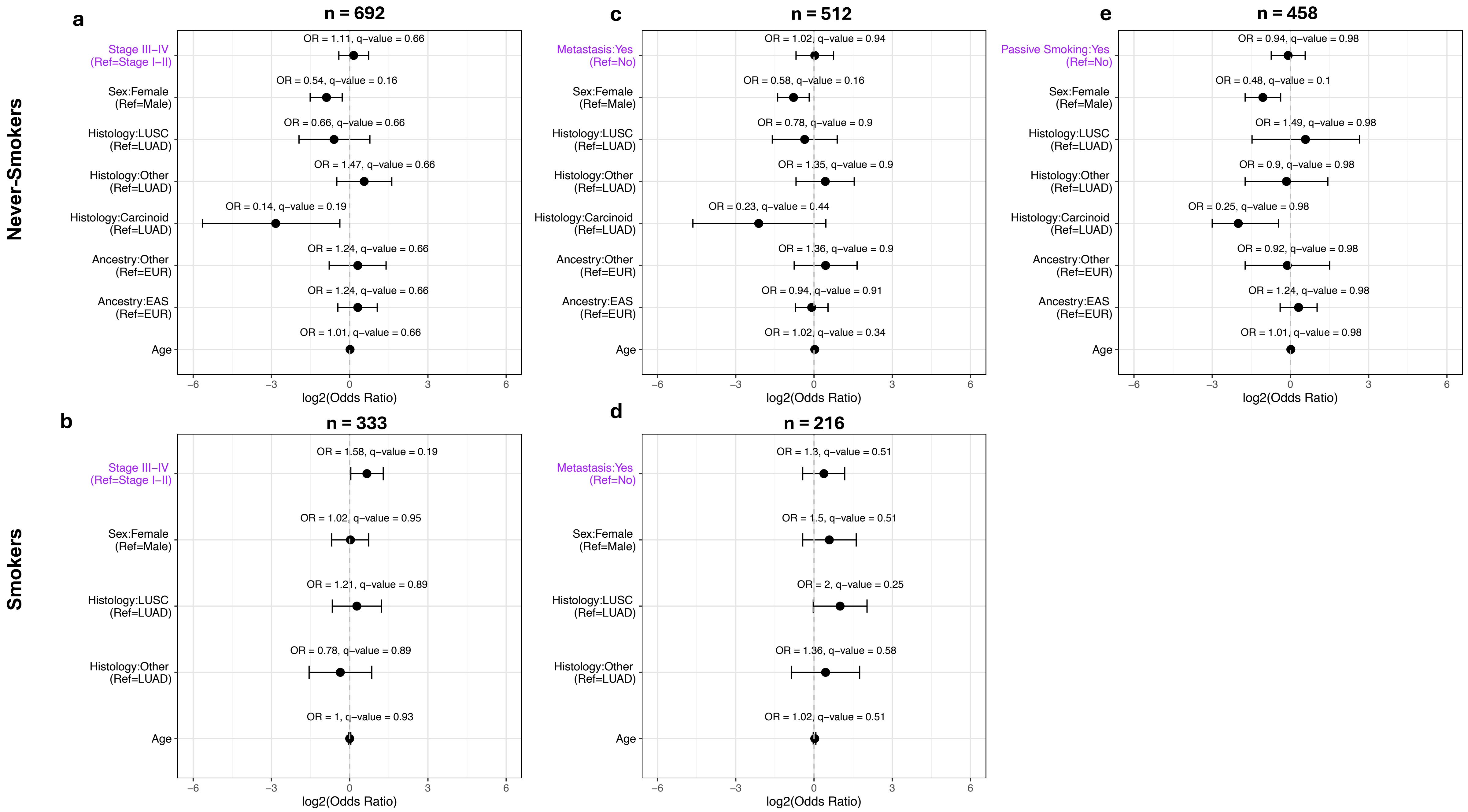

Supplementary Data Fig. 4

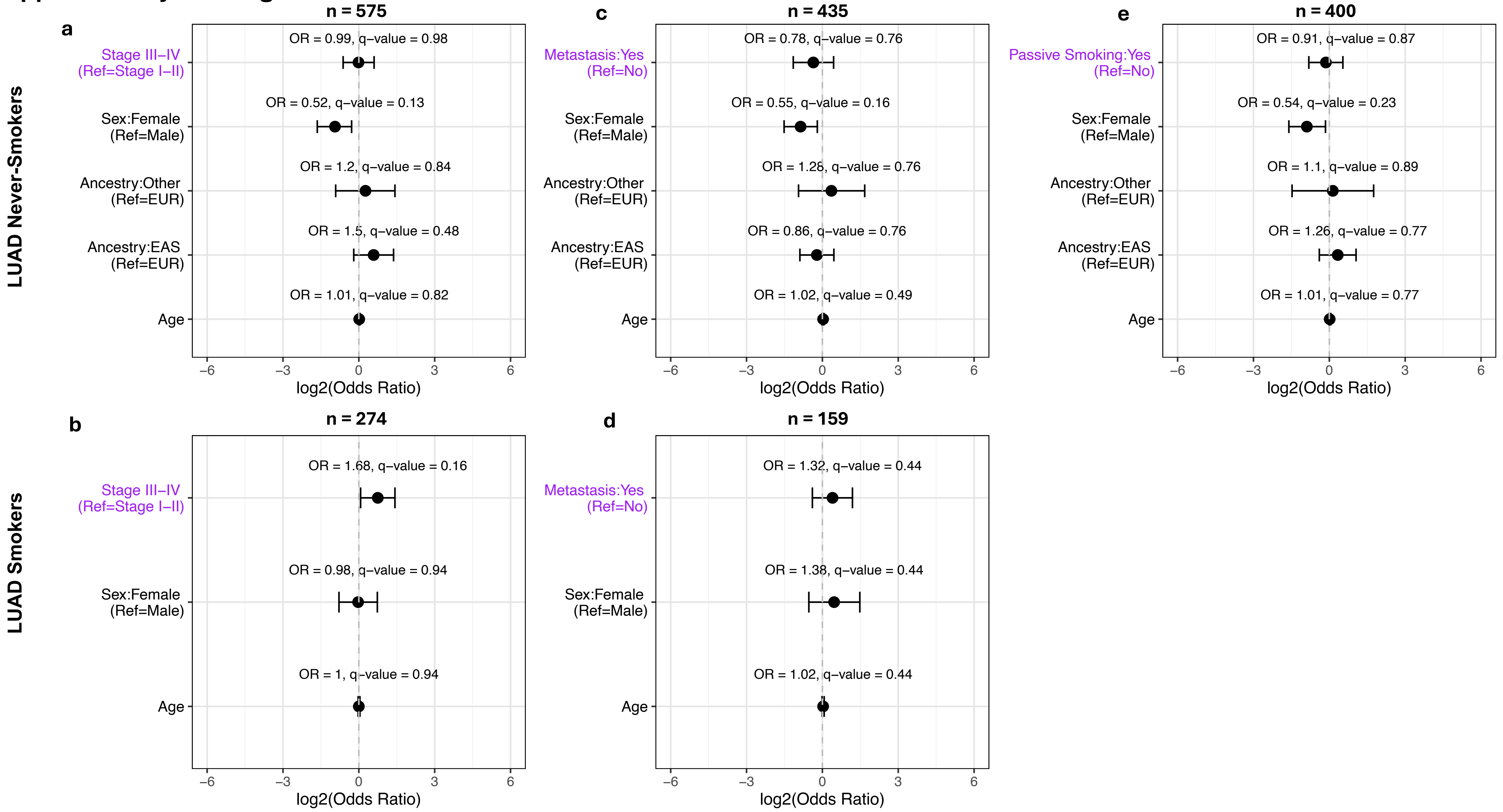

Supplementary Data Fig. 5

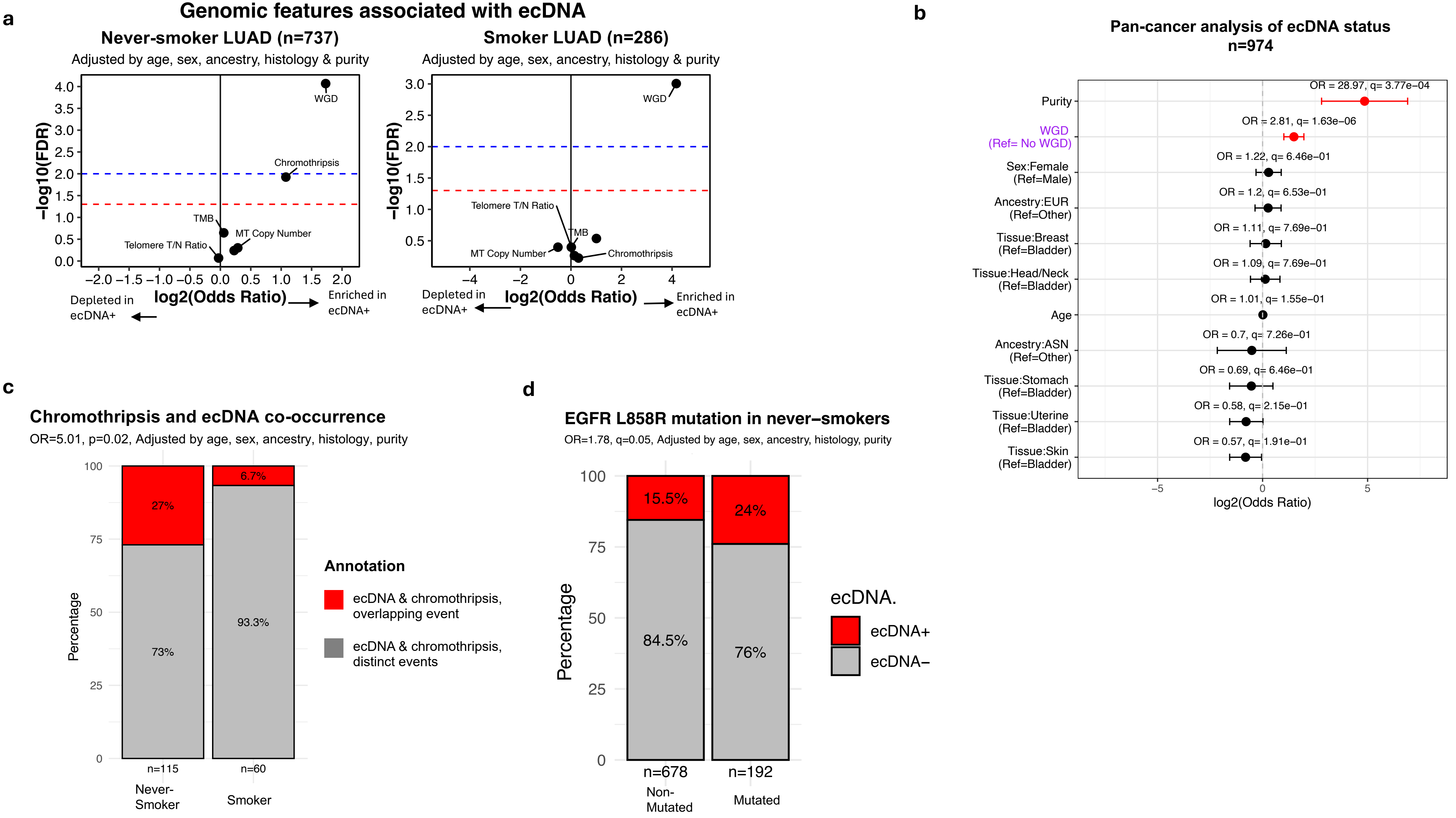

Supplementary Data Fig. 6

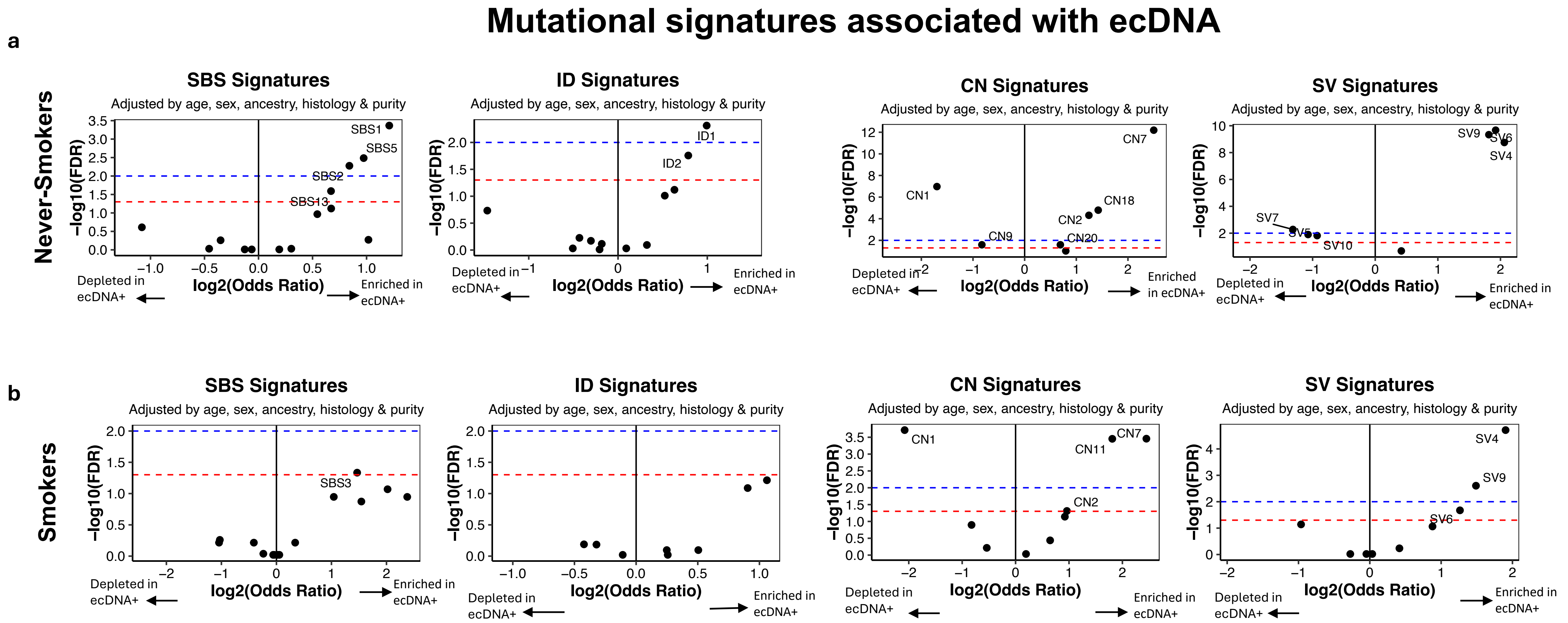

Supplementary Data Fig. 7

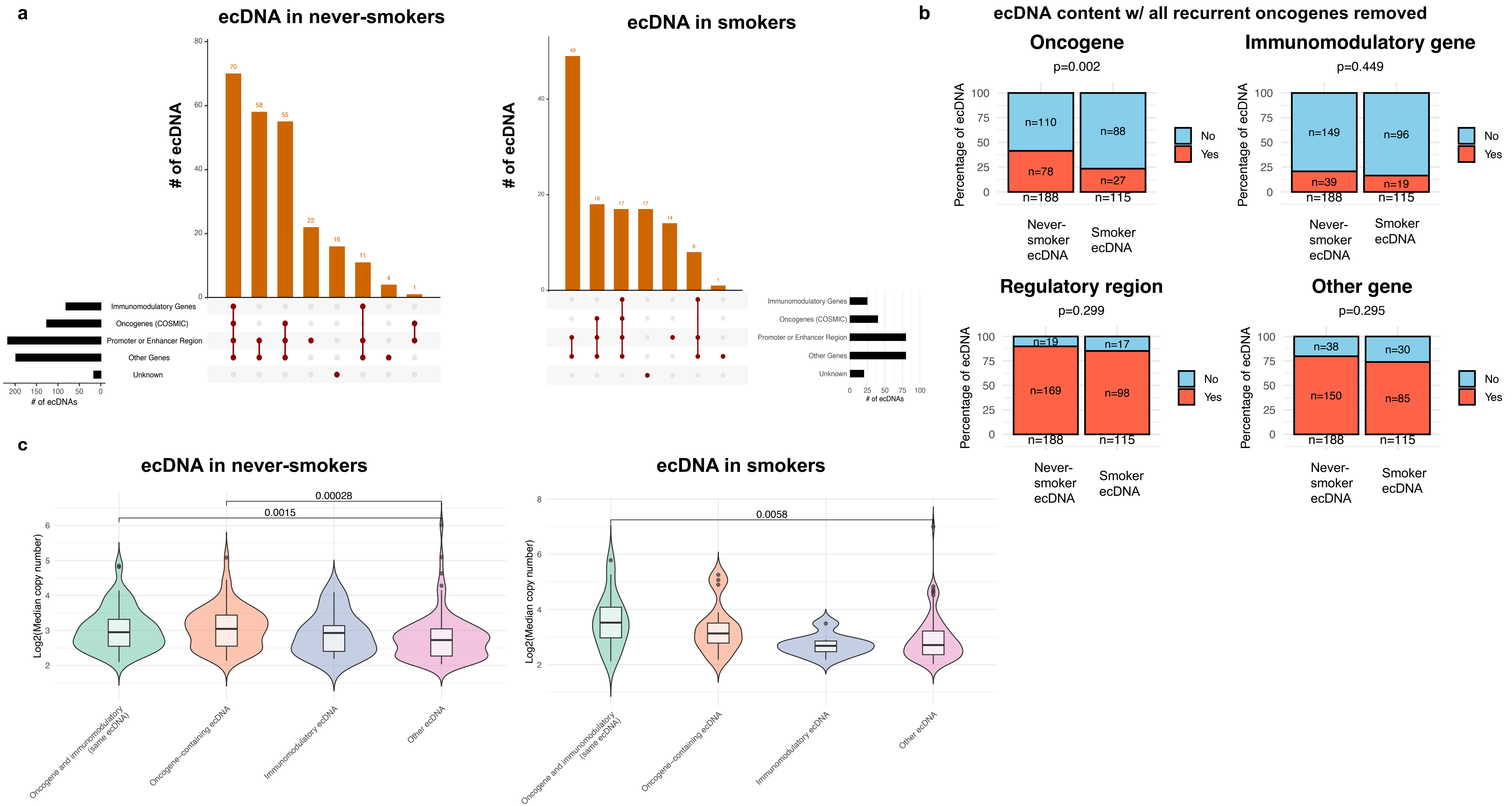

Supplementary Data Fig. 8

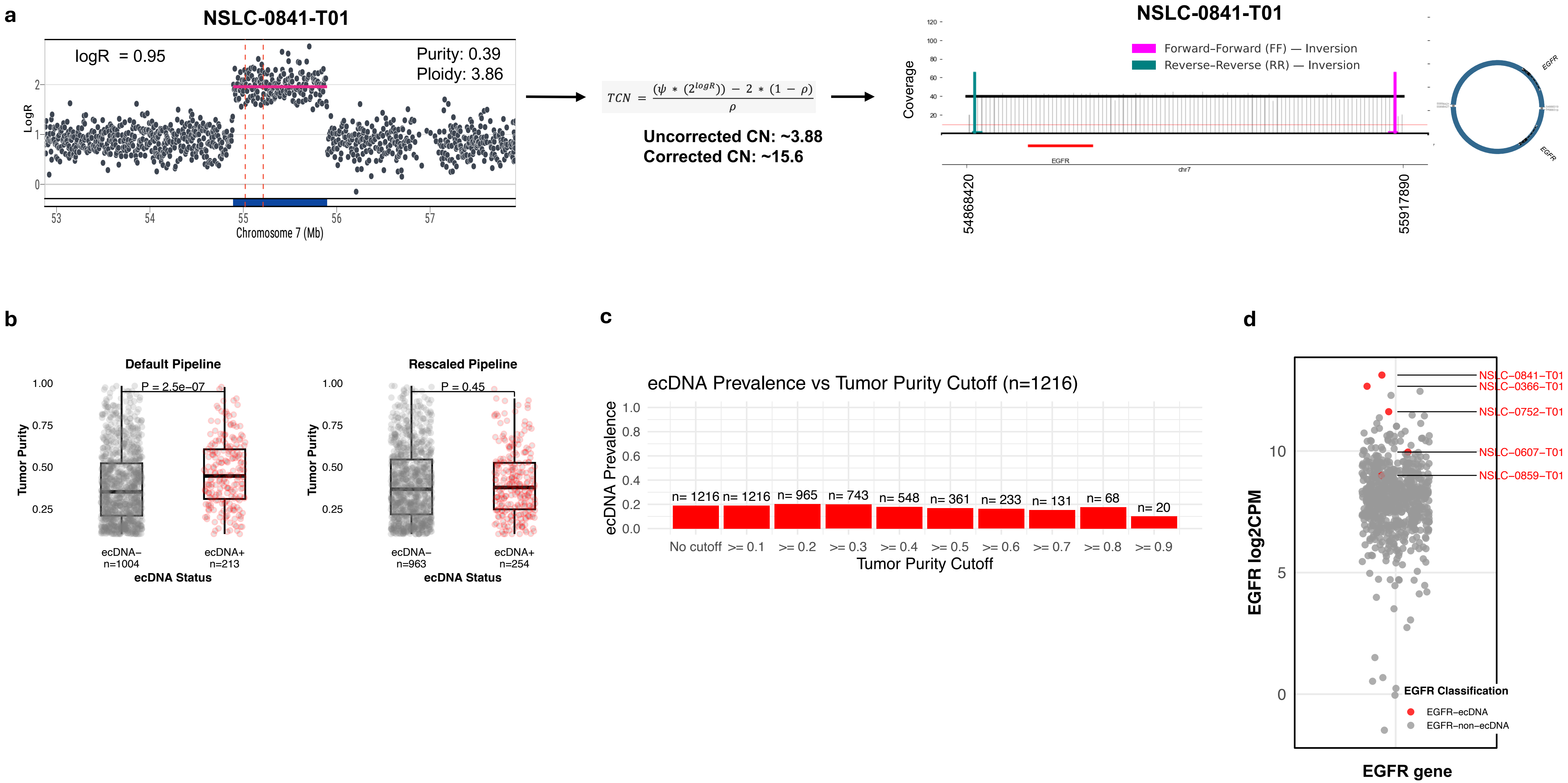

Supplement: Supplementary file 1 [file NIHPP2025.06.03.657117v1-supplement-2.pdf]
